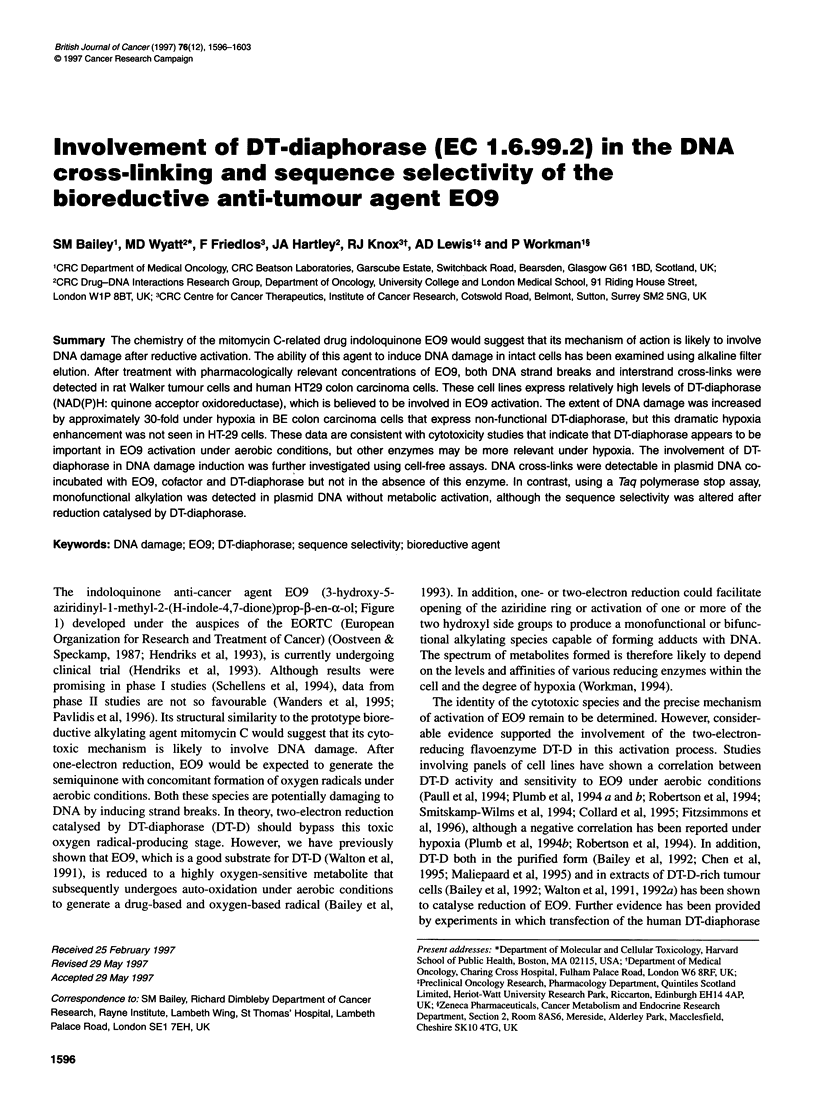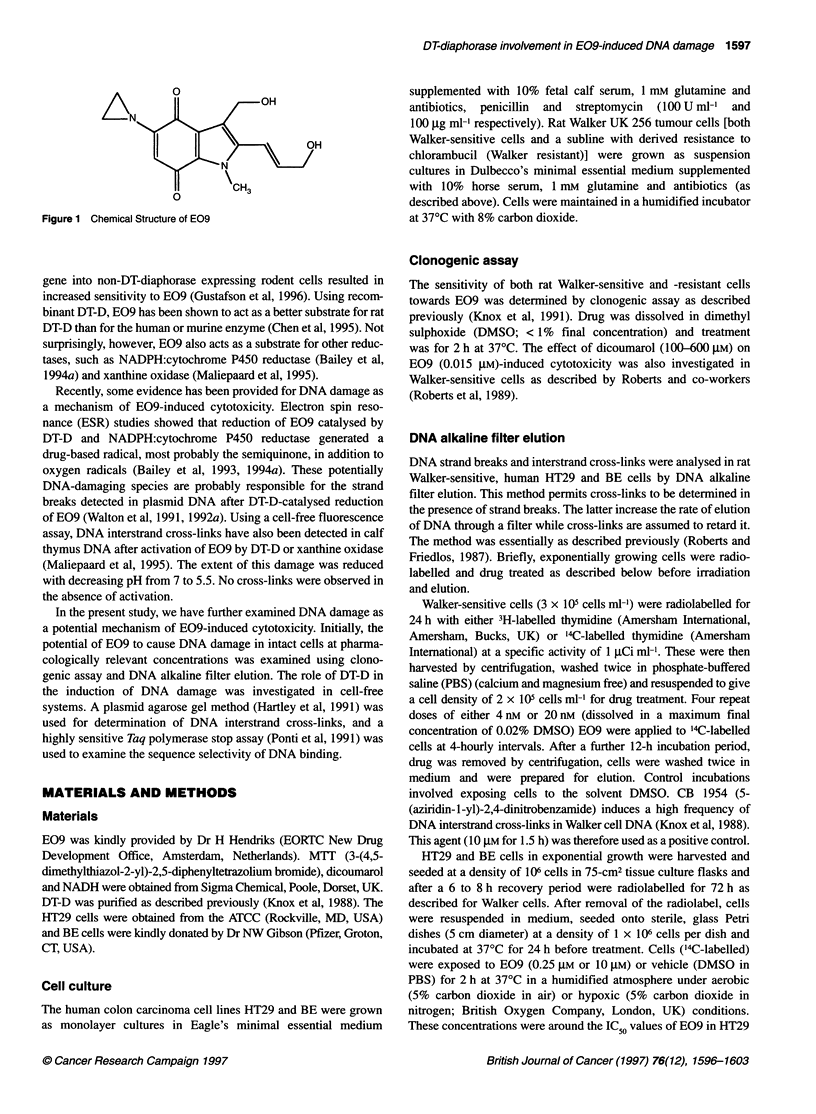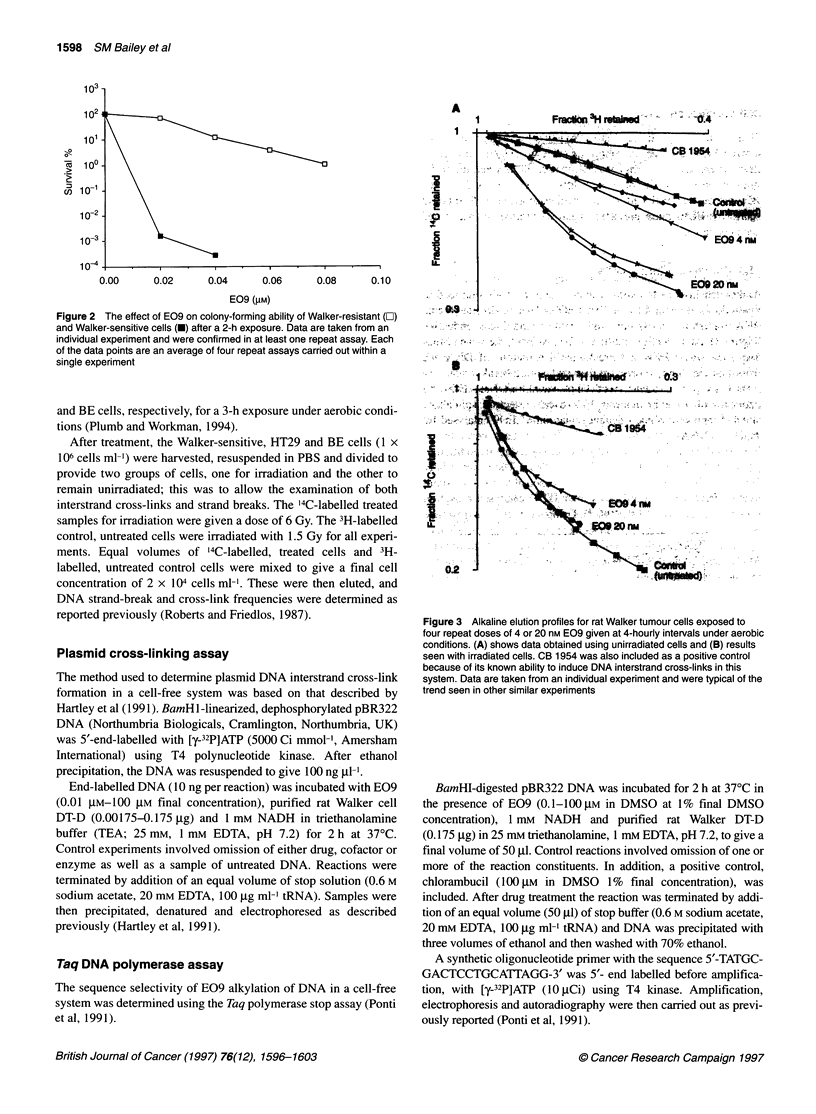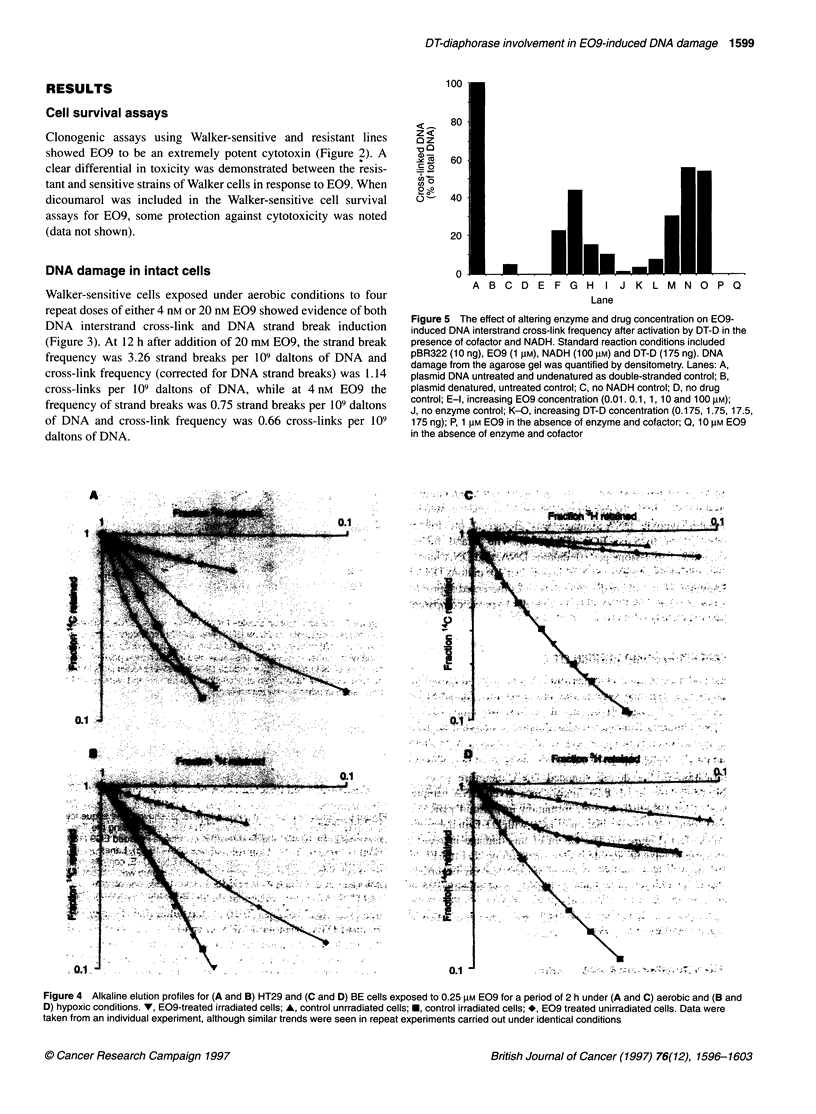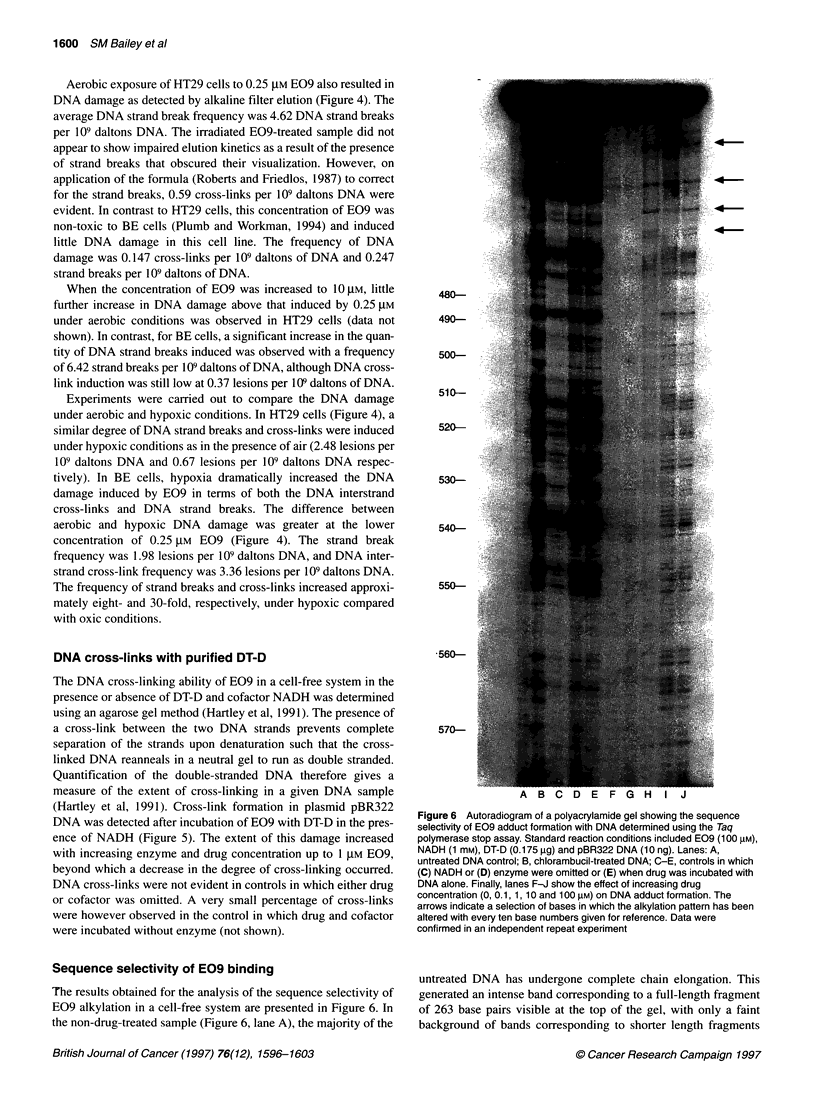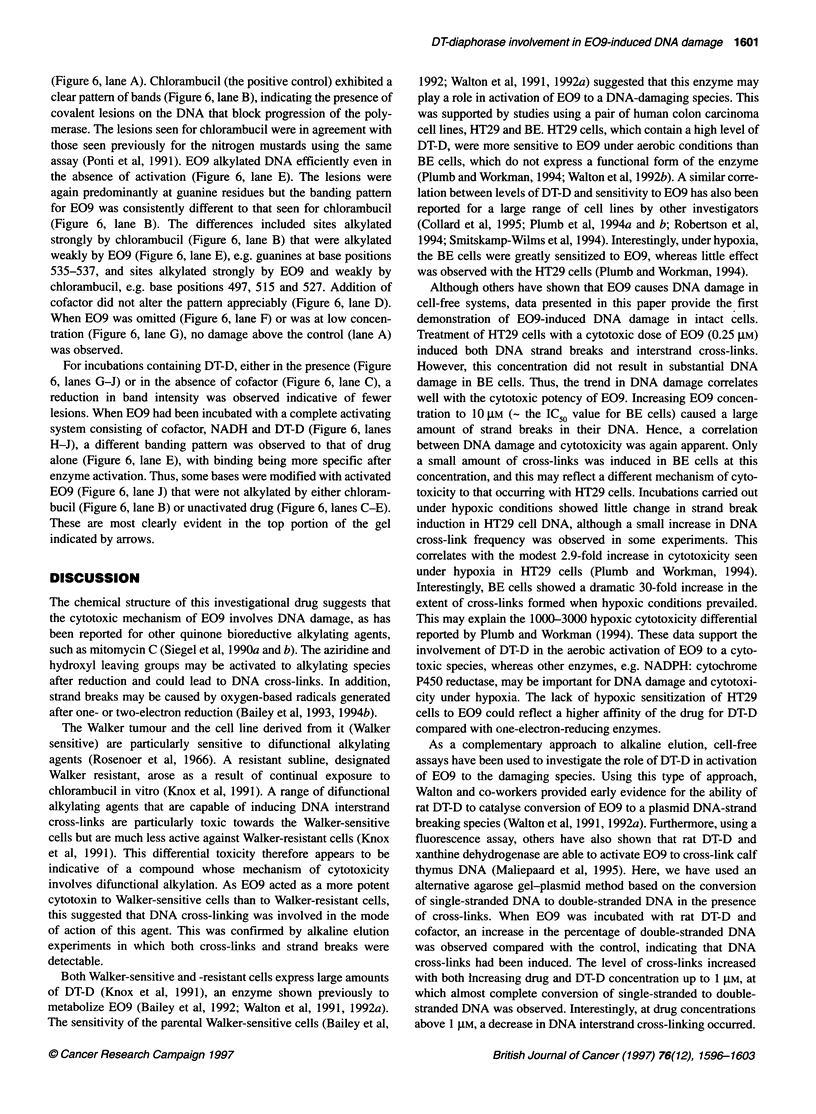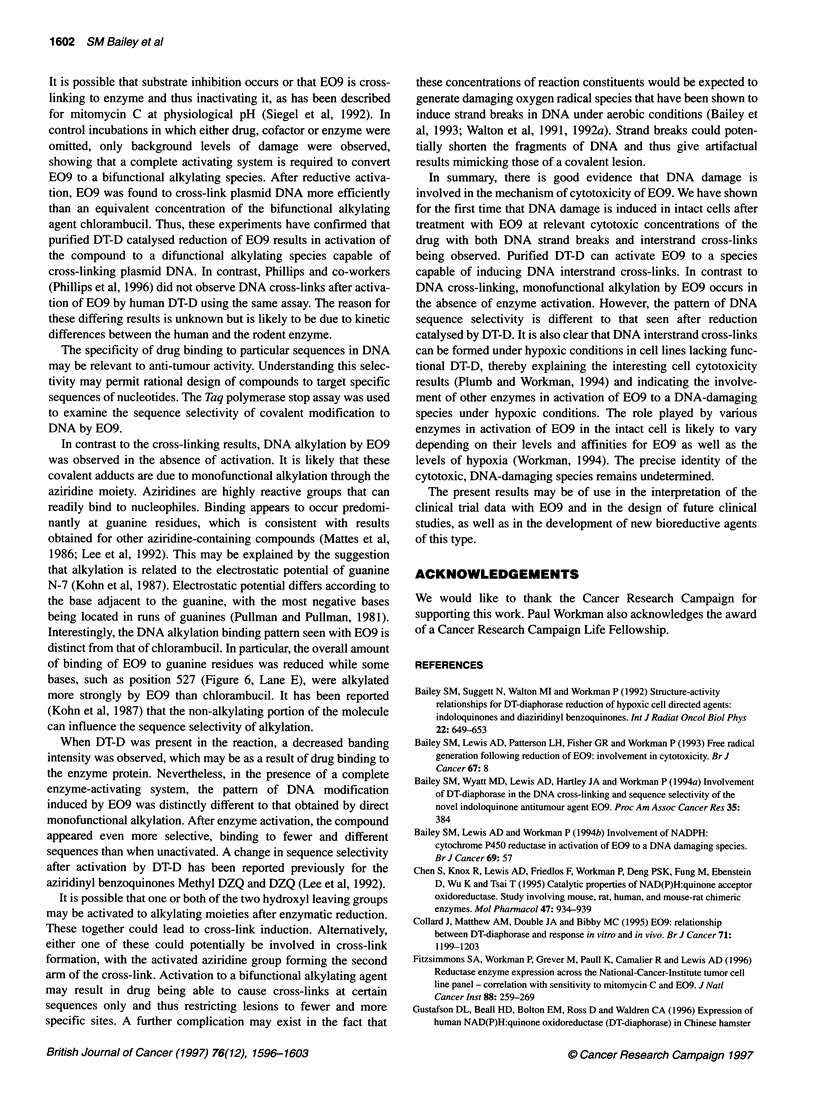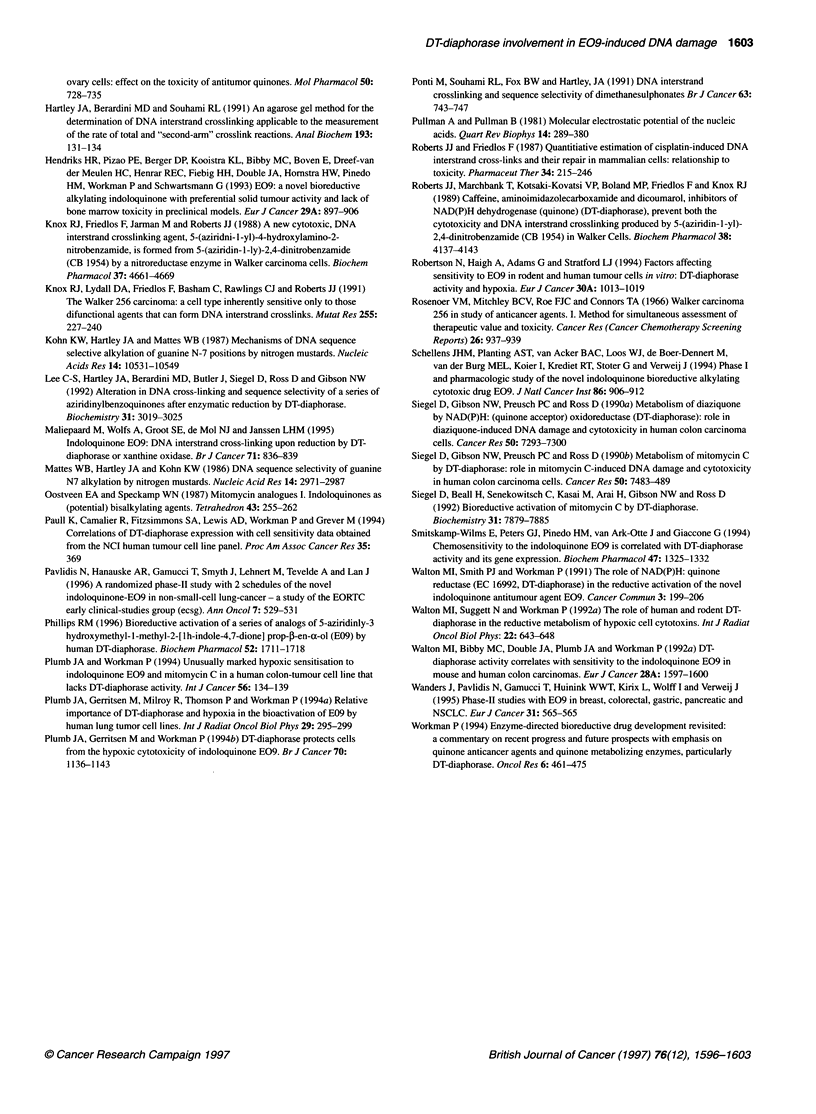# Involvement of DT-diaphorase (EC 1.6.99.2) in the DNA cross-linking and sequence selectivity of the bioreductive anti-tumour agent EO9.

**DOI:** 10.1038/bjc.1997.603

**Published:** 1997

**Authors:** S. M. Bailey, M. D. Wyatt, F. Friedlos, J. A. Hartley, R. J. Knox, A. D. Lewis, P. Workman

**Affiliations:** CRC Department of Medical Oncology, CRC Beatson Laboratories, Glasgow, Scotland, UK.

## Abstract

**Images:**


British Journal of Cancer (1997) 76(12), 1596–1603
© 1997 Cancer Research Campaign

### Involvement of DT-diaphorase (EC 1.6.99.2) in the DNA cross-linking and sequence selectivity of the bioreductive anti-tumour agent EO9

**SM Bailey[1], MD Wyatt[2*], F Friedlos[3], JA Hartley[2], RJ Knox[3†], AD Lewis[1‡] and P Workman[1§]**

[1]CRC Department of Medical Oncology, CRC Beatson Laboratories, Garscube Estate, Switchback Road, Bearsden, Glasgow G61 1BD, Scotland, UK; [2]CRC Drug–DNA Interactions Research Group, Department of Oncology, University College and London Medical School, 91 Riding House Street, London W1P 8BT, UK; [3]CRC Centre for Cancer Therapeutics, Institute of Cancer Research, Cotswold Road, Belmont, Sutton, Surrey SM2 5NG, UK

**Summary** The chemistry of the mitomycin C-related drug indoloquinone EO9 would suggest that its mechanism of action is likely to involve DNA damage after reductive activation. The ability of this agent to induce DNA damage in intact cells has been examined using alkaline filter elution. After treatment with pharmacologically relevant concentrations of EO9, both DNA strand breaks and interstrand cross-links were detected in rat Walker tumour cells and human HT29 colon carcinoma cells. These cell lines express relatively high levels of DT-diaphorase (NAD(P)H: quinone acceptor oxidoreductase), which is believed to be involved in EO9 activation. The extent of DNA damage was increased by approximately 30-fold under hypoxia in BE colon carcinoma cells that express non-functional DT-diaphorase, but this dramatic hypoxia enhancement was not seen in HT-29 cells. These data are consistent with cytotoxicity studies that indicate that DT-diaphorase appears to be important in EO9 activation under aerobic conditions, but other enzymes may be more relevant under hypoxia. The involvement of DT-diaphorase in DNA damage induction was further investigated using cell-free assays. DNA cross-links were detectable in plasmid DNA co-incubated with EO9, cofactor and DT-diaphorase but not in the absence of this enzyme. In contrast, using a *Taq* polymerase stop assay, monofunctional alkylation was detected in plasmid DNA without metabolic activation, although the sequence selectivity was altered after reduction catalysed by DT-diaphorase.

**Keywords:** DNA damage; EO9; DT-diaphorase; sequence selectivity; bioreductive agent

The indoloquinone anti-cancer agent EO9 (3-hydroxy-5-aziridinyl-1-methyl-2-(H-indole-4,7-dione)prop-β-en-α-ol; Figure 1) developed under the auspices of the EORTC (European Organization for Research and Treatment of Cancer) (Oostveen & Speckamp, 1987; Hendriks et al, 1993), is currently undergoing clinical trial (Hendriks et al, 1993). Although results were promising in phase I studies (Schellens et al, 1994), data from phase II studies are not so favourable (Wanders et al, 1995; Pavlidis et al, 1996). Its structural similarity to the prototype bioreductive alkylating agent mitomycin C would suggest that its cytotoxic mechanism is likely to involve DNA damage. After one-electron reduction, EO9 would be expected to generate the semiquinone with concomitant formation of oxygen radicals under aerobic conditions. Both these species are potentially damaging to DNA by inducing strand breaks. In theory, two-electron reduction catalysed by DT-diaphorase (DT-D) should bypass this toxic oxygen radical-producing stage. However, we have previously shown that EO9, which is a good substrate for DT-D (Walton et al, 1991), is reduced to a highly oxygen-sensitive metabolite that subsequently undergoes auto-oxidation under aerobic conditions to generate a drug-based and oxygen-based radical (Bailey et al, 1993). In addition, one- or two-electron reduction could facilitate opening of the aziridine ring or activation of one or more of the two hydroxyl side groups to produce a monofunctional or bifunctional alkylating species capable of forming adducts with DNA. The spectrum of metabolites formed is therefore likely to depend on the levels and affinities of various reducing enzymes within the cell and the degree of hypoxia (Workman, 1994).

The identity of the cytotoxic species and the precise mechanism of activation of EO9 remain to be determined. However, considerable evidence supported the involvement of the two-electron-reducing flavoenzyme DT-D in this activation process. Studies involving panels of cell lines have shown a correlation between DT-D activity and sensitivity to EO9 under aerobic conditions (Paull et al, 1994; Plumb et al, 1994 *a* and *b*; Robertson et al, 1994; Smitskamp-Wilms et al, 1994; Collard et al, 1995; Fitzsimmons et al, 1996), although a negative correlation has been reported under hypoxia (Plumb et al, 1994*b*; Robertson et al, 1994). In addition, DT-D both in the purified form (Bailey et al, 1992; Chen et al, 1995; Maliepaard et al, 1995) and in extracts of DT-D-rich tumour cells (Bailey et al, 1992; Walton et al, 1991, 1992*a*) has been shown to catalyse reduction of EO9. Further evidence has been provided by experiments in which transfection of the human DT-diaphorase

*Received 25 February 1997*
*Revised 29 May 1997*
*Accepted 29 May 1997*

*Correspondence to:* SM Bailey, Richard Dimbleby Department of Cancer Research, Rayne Institute, Lambeth Wing, St Thomas' Hospital, Lambeth Palace Road, London SE1 7EH, UK

*Present addresses:* *Department of Molecular and Cellular Toxicology, Harvard School of Public Health, Boston, MA 02115, USA; †Department of Medical Oncology, Charing Cross Hospital, Fulham Palace Road, London W6 8RF, UK; ‡Preclinical Oncology Research, Pharmacology Department, Quintiles Scotland Limited, Heriot-Watt University Research Park, Riccarton, Edinburgh EH14 4AP, UK; §Zeneca Pharmaceuticals, Cancer Metabolism and Endocrine Research Department, Section 2, Room 8AS6, Mereside, Alderley Park, Macclesfield, Cheshire SK10 4TG, UK

**Figure 1** Chemical Structure of EO9

gene into non-DT-diaphorase expressing rodent cells resulted in increased sensitivity to EO9 (Gustafson et al, 1996). Using recombinant DT-D, EO9 has been shown to act as a better substrate for rat DT-D than for the human or murine enzyme (Chen et al, 1995). Not surprisingly, however, EO9 also acts as a substrate for other reductases, such as NADPH:cytochrome P450 reductase (Bailey et al, 1994*a*) and xanthine oxidase (Maliepaard et al, 1995).

Recently, some evidence has been provided for DNA damage as a mechanism of EO9-induced cytotoxicity. Electron spin resonance (ESR) studies showed that reduction of EO9 catalysed by DT-D and NADPH:cytochrome P450 reductase generated a drug-based radical, most probably the semiquinone, in addition to oxygen radicals (Bailey et al, 1993, 1994*a*). These potentially DNA-damaging species are probably responsible for the strand breaks detected in plasmid DNA after DT-D-catalysed reduction of EO9 (Walton et al, 1991, 1992*a*). Using a cell-free fluorescence assay, DNA interstrand cross-links have also been detected in calf thymus DNA after activation of EO9 by DT-D or xanthine oxidase (Maliepaard et al, 1995). The extent of this damage was reduced with decreasing pH from 7 to 5.5. No cross-links were observed in the absence of activation.

In the present study, we have further examined DNA damage as a potential mechanism of EO9-induced cytotoxicity. Initially, the potential of EO9 to cause DNA damage in intact cells at pharmacologically relevant concentrations was examined using clonogenic assay and DNA alkaline filter elution. The role of DT-D in the induction of DNA damage was investigated in cell-free systems. A plasmid agarose gel method (Hartley et al, 1991) was used for determination of DNA interstrand cross-links, and a highly sensitive *Taq* polymerase stop assay (Ponti et al, 1991) was used to examine the sequence selectivity of DNA binding.

### MATERIALS AND METHODS

### Materials

EO9 was kindly provided by Dr H Hendriks (EORTC New Drug Development Office, Amsterdam, Netherlands). MTT (3-(4,5-dimethylthiazol-2-yl)-2,5-diphenyltetrazolium bromide), dicoumarol and NADH were obtained from Sigma Chemical, Poole, Dorset, UK. DT-D was purified as described previously (Knox et al, 1988). The HT29 cells were obtained from the ATCC (Rockville, MD, USA) and BE cells were kindly donated by Dr NW Gibson (Pfizer, Groton, CT, USA).

### Cell culture

The human colon carcinoma cell lines HT29 and BE were grown as monolayer cultures in Eagle's minimal essential medium supplemented with 10% fetal calf serum, 1 mM glutamine and antibiotics, penicillin and streptomycin (100 U $ml^{-1}$ and 100 µg $ml^{-1}$ respectively). Rat Walker UK 256 tumour cells [both Walker-sensitive cells and a subline with derived resistance to chlorambucil (Walker resistant)] were grown as suspension cultures in Dulbecco's minimal essential medium supplemented with 10% horse serum, 1 mM glutamine and antibiotics (as described above). Cells were maintained in a humidified incubator at 37°C with 8% carbon dioxide.

### Clonogenic assay

The sensitivity of both rat Walker-sensitive and -resistant cells towards EO9 was determined by clonogenic assay as described previously (Knox et al, 1991). Drug was dissolved in dimethyl sulphoxide (DMSO; < 1% final concentration) and treatment was for 2 h at 37°C. The effect of dicoumarol (100–600 µM) on EO9 (0.015 µM)-induced cytotoxicity was also investigated in Walker-sensitive cells as described by Roberts and co-workers (Roberts et al, 1989).

### DNA alkaline filter elution

DNA strand breaks and interstrand cross-links were analysed in rat Walker-sensitive, human HT29 and BE cells by DNA alkaline filter elution. This method permits cross-links to be determined in the presence of strand breaks. The latter increase the rate of elution of DNA through a filter while cross-links are assumed to retard it. The method was essentially as described previously (Roberts and Friedlos, 1987). Briefly, exponentially growing cells were radiolabelled and drug treated as described below before irradiation and elution.

Walker-sensitive cells ($3 \times 10^5$ cells $ml^{-1}$) were radiolabelled for 24 h with either $^3$H-labelled thymidine (Amersham International, Amersham, Bucks, UK) or $^{14}$C-labelled thymidine (Amersham International) at a specific activity of 1 µCi $ml^{-1}$. These were then harvested by centrifugation, washed twice in phosphate-buffered saline (PBS) (calcium and magnesium free) and resuspended to give a cell density of $2 \times 10^5$ cells $ml^{-1}$ for drug treatment. Four repeat doses of either 4 nM or 20 nM (dissolved in a maximum final concentration of 0.02% DMSO) EO9 were applied to $^{14}$C-labelled cells at 4-hourly intervals. After a further 12-h incubation period, drug was removed by centrifugation, cells were washed twice in medium and were prepared for elution. Control incubations involved exposing cells to the solvent DMSO. CB 1954 (5-(aziridin-1-yl)-2,4-dinitrobenzamide) induces a high frequency of DNA interstrand cross-links in Walker cell DNA (Knox et al, 1988). This agent (10 µM for 1.5 h) was therefore used as a positive control.

HT29 and BE cells in exponential growth were harvested and seeded at a density of $10^6$ cells in 75-$cm^2$ tissue culture flasks and after a 6 to 8 h recovery period were radiolabelled for 72 h as described for Walker cells. After removal of the radiolabel, cells were resuspended in medium, seeded onto sterile, glass Petri dishes (5 cm diameter) at a density of $1 \times 10^6$ cells per dish and incubated at 37°C for 24 h before treatment. Cells ($^{14}$C-labelled) were exposed to EO9 (0.25 µM or 10 µM) or vehicle (DMSO in PBS) for 2 h at 37°C in a humidified atmosphere under aerobic (5% carbon dioxide in air) or hypoxic (5% carbon dioxide in nitrogen; British Oxygen Company, London, UK) conditions. These concentrations were around the $IC_{50}$ values of EO9 in HT29

© Cancer Research Campaign 1997

**Figure 2** The effect of EO9 on colony-forming ability of Walker-resistant (□) and Walker-sensitive cells (■) after a 2-h exposure. Data are taken from an individual experiment and were confirmed in at least one repeat assay. Each of the data points are an average of four repeat assays carried out within a single experiment and BE cells, respectively, for a 3-h exposure under aerobic conditions (Plumb and Workman, 1994).

After treatment, the Walker-sensitive, HT29 and BE cells ($1 \times 10^6$ cells $ml^{-1}$) were harvested, resuspended in PBS and divided to provide two groups of cells, one for irradiation and the other to remain unirradiated; this was to allow the examination of both interstrand cross-links and strand breaks. The $^{14}C$-labelled treated samples for irradiation were given a dose of 6 Gy. The $^3H$-labelled control, untreated cells were irradiated with 1.5 Gy for all experiments. Equal volumes of $^{14}C$-labelled, treated cells and $^3H$-labelled, untreated control cells were mixed to give a final cell concentration of $2 \times 10^4$ cells $ml^{-1}$. These were then eluted, and DNA strand-break and cross-link frequencies were determined as reported previously (Roberts and Friedlos, 1987).

### Plasmid cross-linking assay

The method used to determine plasmid DNA interstrand cross-link formation in a cell-free system was based on that described by Hartley et al (1991). *Bam*H1-linearized, dephosphorylated pBR322 DNA (Northumbria Biologicals, Cramlington, Northumbria, UK) was 5′-end-labelled with [γ-$^{32}$P]ATP (5000 Ci $mmol^{-1}$, Amersham International) using T4 polynucleotide kinase. After ethanol precipitation, the DNA was resuspended to give 100 ng $\mu l^{-1}$.

End-labelled DNA (10 ng per reaction) was incubated with EO9 (0.01 μM–100 μM final concentration), purified rat Walker cell DT-D (0.00175–0.175 μg) and 1 mM NADH in triethanolamine buffer (TEA; 25 mM, 1 mM EDTA, pH 7.2) for 2 h at 37°C. Control experiments involved omission of either drug, cofactor or enzyme as well as a sample of untreated DNA. Reactions were terminated by addition of an equal volume of stop solution (0.6 M sodium acetate, 20 mM EDTA, 100 μg $ml^{-1}$ tRNA). Samples were then precipitated, denatured and electrophoresed as described previously (Hartley et al, 1991).

### *Taq* DNA polymerase assay

The sequence selectivity of EO9 alkylation of DNA in a cell-free system was determined using the *Taq* polymerase stop assay (Ponti et al, 1991).

**Figure 3** Alkaline elution profiles for rat Walker tumour cells exposed to four repeat doses of 4 or 20 nM EO9 given at 4-hourly intervals under aerobic conditions. (**A**) shows data obtained using unirradiated cells and (**B**) results seen with irradiated cells. CB 1954 was also included as a positive control because of its known ability to induce DNA interstrand cross-links in this system. Data are taken from an individual experiment and were typical of the trend seen in other similar experiments

*Bam*HI-digested pBR322 DNA was incubated for 2 h at 37°C in the presence of EO9 (0.1–100 μM in DMSO at 1% final DMSO concentration), 1 mM NADH and purified rat Walker DT-D (0.175 μg) in 25 mM triethanolamine, 1 mM EDTA, pH 7.2, to give a final volume of 50 μl. Control reactions involved omission of one or more of the reaction constituents. In addition, a positive control, chlorambucil (100 μM in DMSO 1% final concentration), was included. After drug treatment the reaction was terminated by addition of an equal volume (50 μl) of stop buffer (0.6 M sodium acetate, 20 mM EDTA, 100 μg $ml^{-1}$ tRNA) and DNA was precipitated with three volumes of ethanol and then washed with 70% ethanol.

A synthetic oligonucleotide primer with the sequence 5′-TATGC-GACTCCTGCATTAGG-3′ was 5′- end labelled before amplification, with [γ-$^{32}$P]ATP (10 μCi) using T4 kinase. Amplification, electrophoresis and autoradiography were then carried out as previously reported (Ponti et al, 1991).

© Cancer Research Campaign 1997

### RESULTS

### Cell survival assays

Clonogenic assays using Walker-sensitive and resistant lines showed EO9 to be an extremely potent cytotoxin (Figure 2). A clear differential in toxicity was demonstrated between the resistant and sensitive strains of Walker cells in response to EO9. When dicoumarol was included in the Walker-sensitive cell survival assays for EO9, some protection against cytotoxicity was noted (data not shown).

### DNA damage in intact cells

Walker-sensitive cells exposed under aerobic conditions to four repeat doses of either 4 nM or 20 nM EO9 showed evidence of both DNA interstrand cross-link and DNA strand break induction (Figure 3). At 12 h after addition of 20 mM EO9, the strand break frequency was 3.26 strand breaks per $10^9$ daltons of DNA and cross-link frequency (corrected for DNA strand breaks) was 1.14 cross-links per $10^9$ daltons of DNA, while at 4 nM EO9 the frequency of strand breaks was 0.75 strand breaks per $10^9$ daltons of DNA and cross-link frequency was 0.66 cross-links per $10^9$ daltons of DNA.

**Figure 5** The effect of altering enzyme and drug concentration on EO9-induced DNA interstrand cross-link frequency after activation by DT-D in the presence of cofactor and NADH. Standard reaction conditions included pBR322 (10 ng), EO9 (1 μM), NADH (100 μM) and DT-D (175 ng). DNA damage from the agarose gel was quantified by densitometry. Lanes: A, plasmid DNA untreated and undenatured as double-stranded control; B, plasmid denatured, untreated control; C, no NADH control; D, no drug control; E–I, increasing EO9 concentration (0.01. 0.1, 1, 10 and 100 μM); J, no enzyme control; K–O, increasing DT-D concentration (0.175, 1.75, 17.5, 175 ng); P, 1 μM EO9 in the absence of enzyme and cofactor; Q, 10 μM EO9 in the absence of enzyme and cofactor

**Figure 4** Alkaline elution profiles for (**A** and **B**) HT29 and (**C** and **D**) BE cells exposed to 0.25 μM EO9 for a period of 2 h under (**A** and **C**) aerobic and (**B** and **D**) hypoxic conditions. ▼, EO9-treated irradiated cells; ▲, control unrradiated cells; ■, control irradiated cells; ◆, EO9 treated unirradiated cells. Data were taken from an individual experiment, although similar trends were seen in repeat experiments carried out under identical conditions

Aerobic exposure of HT29 cells to 0.25 μM EO9 also resulted in DNA damage as detected by alkaline filter elution (Figure 4). The average DNA strand break frequency was 4.62 DNA strand breaks per $10^9$ daltons DNA. The irradiated EO9-treated sample did not appear to show impaired elution kinetics as a result of the presence of strand breaks that obscured their visualization. However, on application of the formula (Roberts and Friedlos, 1987) to correct for the strand breaks, 0.59 cross-links per $10^9$ daltons DNA were evident. In contrast to HT29 cells, this concentration of EO9 was non-toxic to BE cells (Plumb and Workman, 1994) and induced little DNA damage in this cell line. The frequency of DNA damage was 0.147 cross-links per $10^9$ daltons of DNA and 0.247 strand breaks per $10^9$ daltons of DNA.

When the concentration of EO9 was increased to 10 μM, little further increase in DNA damage above that induced by 0.25 μM under aerobic conditions was observed in HT29 cells (data not shown). In contrast, for BE cells, a significant increase in the quantity of DNA strand breaks induced was observed with a frequency of 6.42 strand breaks per $10^9$ daltons of DNA, although DNA cross-link induction was still low at 0.37 lesions per $10^9$ daltons of DNA.

Experiments were carried out to compare the DNA damage under aerobic and hypoxic conditions. In HT29 cells (Figure 4), a similar degree of DNA strand breaks and cross-links were induced under hypoxic conditions as in the presence of air (2.48 lesions per $10^9$ daltons DNA and 0.67 lesions per $10^9$ daltons DNA respectively). In BE cells, hypoxia dramatically increased the DNA damage induced by EO9 in terms of both the DNA interstrand cross-links and DNA strand breaks. The difference between aerobic and hypoxic DNA damage was greater at the lower concentration of 0.25 μM EO9 (Figure 4). The strand break frequency was 1.98 lesions per $10^9$ daltons DNA, and DNA interstrand cross-link frequency was 3.36 lesions per $10^9$ daltons DNA. The frequency of strand breaks and cross-links increased approximately eight- and 30-fold, respectively, under hypoxic compared with oxic conditions.

### DNA cross-links with purified DT-D

The DNA cross-linking ability of EO9 in a cell-free system in the presence or absence of DT-D and cofactor NADH was determined using an agarose gel method (Hartley et al, 1991). The presence of a cross-link between the two DNA strands prevents complete separation of the strands upon denaturation such that the cross-linked DNA reanneals in a neutral gel to run as double stranded. Quantification of the double-stranded DNA therefore gives a measure of the extent of cross-linking in a given DNA sample (Hartley et al, 1991). Cross-link formation in plasmid pBR322 DNA was detected after incubation of EO9 with DT-D in the presence of NADH (Figure 5). The extent of this damage increased with increasing enzyme and drug concentration up to 1 μM EO9, beyond which a decrease in the degree of cross-linking occurred. DNA cross-links were not evident in controls in which either drug or cofactor was omitted. A very small percentage of cross-links were however observed in the control in which drug and cofactor were incubated without enzyme (not shown).

### Sequence selectivity of EO9 binding

The results obtained for the analysis of the sequence selectivity of EO9 alkylation in a cell-free system are presented in Figure 6. In the non-drug-treated sample (Figure 6, lane A), the majority of the untreated DNA has undergone complete chain elongation. This generated an intense band corresponding to a full-length fragment of 263 base pairs visible at the top of the gel, with only a faint background of bands corresponding to shorter length fragments

**Figure 6** Autoradiogram of a polyacrylamide gel showing the sequence selectivity of EO9 adduct formation with DNA determined using the *Taq* polymerase stop assay. Standard reaction conditions included EO9 (100 μM), NADH (1 mM), DT-D (0.175 μg) and pBR322 DNA (10 ng). Lanes: A, untreated DNA control; B, chlorambucil-treated DNA; C–E, controls in which (**C**) NADH or (**D**) enzyme were omitted or (**E**) when drug was incubated with DNA alone. Finally, lanes F–J show the effect of increasing drug concentration (0, 0.1, 1, 10 and 100 μM) on DNA adduct formation. The arrows indicate a selection of bases in which the alkylation pattern has been altered with every ten base numbers given for reference. Data were confirmed in an independent repeat experiment

© Cancer Research Campaign 1997

(Figure 6, lane A). Chlorambucil (the positive control) exhibited a clear pattern of bands (Figure 6, lane B), indicating the presence of covalent lesions on the DNA that block progression of the polymerase. The lesions seen for chlorambucil were in agreement with those seen previously for the nitrogen mustards using the same assay (Ponti et al, 1991). EO9 alkylated DNA efficiently even in the absence of activation (Figure 6, lane E). The lesions were again predominantly at guanine residues but the banding pattern for EO9 was consistently different to that seen for chlorambucil (Figure 6, lane B). The differences included sites alkylated strongly by chlorambucil (Figure 6, lane B) that were alkylated weakly by EO9 (Figure 6, lane E), e.g. guanines at base positions 535–537, and sites alkylated strongly by EO9 and weakly by chlorambucil, e.g. base positions 497, 515 and 527. Addition of cofactor did not alter the pattern appreciably (Figure 6, lane D). When EO9 was omitted (Figure 6, lane F) or was at low concentration (Figure 6, lane G), no damage above the control (lane A) was observed.

For incubations containing DT-D, either in the presence (Figure 6, lanes G–J) or in the absence of cofactor (Figure 6, lane C), a reduction in band intensity was observed indicative of fewer lesions. When EO9 had been incubated with a complete activating system consisting of cofactor, NADH and DT-D (Figure 6, lanes H–J), a different banding pattern was observed to that of drug alone (Figure 6, lane E), with binding being more specific after enzyme activation. Thus, some bases were modified with activated EO9 (Figure 6, lane J) that were not alkylated by either chlorambucil (Figure 6, lane B) or unactivated drug (Figure 6, lanes C–E). These are most clearly evident in the top portion of the gel indicated by arrows.

### DISCUSSION

The chemical structure of this investigational drug suggests that the cytotoxic mechanism of EO9 involves DNA damage, as has been reported for other quinone bioreductive alkylating agents, such as mitomycin C (Siegel et al, 1990*a* and *b*). The aziridine and hydroxyl leaving groups may be activated to alkylating species after reduction and could lead to DNA cross-links. In addition, strand breaks may be caused by oxygen-based radicals generated after one- or two-electron reduction (Bailey et al, 1993, 1994*b*).

The Walker tumour and the cell line derived from it (Walker sensitive) are particularly sensitive to difunctional alkylating agents (Rosenoer et al, 1966). A resistant subline, designated Walker resistant, arose as a result of continual exposure to chlorambucil in vitro (Knox et al, 1991). A range of difunctional alkylating agents that are capable of inducing DNA interstrand cross-links are particularly toxic towards the Walker-sensitive cells but are much less active against Walker-resistant cells (Knox et al, 1991). This differential toxicity therefore appears to be indicative of a compound whose mechanism of cytotoxicity involves difunctional alkylation. As EO9 acted as a more potent cytotoxin to Walker-sensitive cells than to Walker-resistant cells, this suggested that DNA cross-linking was involved in the mode of action of this agent. This was confirmed by alkaline elution experiments in which both cross-links and strand breaks were detectable.

Both Walker-sensitive and -resistant cells express large amounts of DT-D (Knox et al, 1991), an enzyme shown previously to metabolize EO9 (Bailey et al, 1992; Walton et al, 1991, 1992*a*). The sensitivity of the parental Walker-sensitive cells (Bailey et al, 1992; Walton et al, 1991, 1992*a*) suggested that this enzyme may play a role in activation of EO9 to a DNA-damaging species. This was supported by studies using a pair of human colon carcinoma cell lines, HT29 and BE. HT29 cells, which contain a high level of DT-D, were more sensitive to EO9 under aerobic conditions than BE cells, which do not express a functional form of the enzyme (Plumb and Workman, 1994; Walton et al, 1992*b*). A similar correlation between levels of DT-D and sensitivity to EO9 has also been reported for a large range of cell lines by other investigators (Collard et al, 1995; Plumb et al, 1994*a* and *b*; Robertson et al, 1994; Smitskamp-Wilms et al, 1994). Interestingly, under hypoxia, the BE cells were greatly sensitized to EO9, whereas little effect was observed with the HT29 cells (Plumb and Workman, 1994).

Although others have shown that EO9 causes DNA damage in cell-free systems, data presented in this paper provide the first demonstration of EO9-induced DNA damage in intact cells. Treatment of HT29 cells with a cytotoxic dose of EO9 (0.25 µM) induced both DNA strand breaks and interstrand cross-links. However, this concentration did not result in substantial DNA damage in BE cells. Thus, the trend in DNA damage correlates well with the cytotoxic potency of EO9. Increasing EO9 concentration to 10 µM (~ the $IC_{50}$ value for BE cells) caused a large amount of strand breaks in their DNA. Hence, a correlation between DNA damage and cytotoxicity was again apparent. Only a small amount of cross-links was induced in BE cells at this concentration, and this may reflect a different mechanism of cytotoxicity to that occurring with HT29 cells. Incubations carried out under hypoxic conditions showed little change in strand break induction in HT29 cell DNA, although a small increase in DNA cross-link frequency was observed in some experiments. This correlates with the modest 2.9-fold increase in cytotoxicity seen under hypoxia in HT29 cells (Plumb and Workman, 1994). Interestingly, BE cells showed a dramatic 30-fold increase in the extent of cross-links formed when hypoxic conditions prevailed. This may explain the 1000–3000 hypoxic cytotoxicity differential reported by Plumb and Workman (1994). These data support the involvement of DT-D in the aerobic activation of EO9 to a cytotoxic species, whereas other enzymes, e.g. NADPH: cytochrome P450 reductase, may be important for DNA damage and cytotoxicity under hypoxia. The lack of hypoxic sensitization of HT29 cells to EO9 could reflect a higher affinity of the drug for DT-D compared with one-electron-reducing enzymes.

As a complementary approach to alkaline elution, cell-free assays have been used to investigate the role of DT-D in activation of EO9 to the damaging species. Using this type of approach, Walton and co-workers provided early evidence for the ability of rat DT-D to catalyse conversion of EO9 to a plasmid DNA-strand breaking species (Walton et al, 1991, 1992*a*). Furthermore, using a fluorescence assay, others have also shown that rat DT-D and xanthine dehydrogenase are able to activate EO9 to cross-link calf thymus DNA (Maliepaard et al, 1995). Here, we have used an alternative agarose gel–plasmid method based on the conversion of single-stranded DNA to double-stranded DNA in the presence of cross-links. When EO9 was incubated with rat DT-D and cofactor, an increase in the percentage of double-stranded DNA was observed compared with the control, indicating that DNA cross-links had been induced. The level of cross-links increased with both increasing drug and DT-D concentration up to 1 µM, at which almost complete conversion of single-stranded to double-stranded DNA was observed. Interestingly, at drug concentrations above 1 µM, a decrease in DNA interstrand cross-linking occurred.

© Cancer Research Campaign 1997

It is possible that substrate inhibition occurs or that EO9 is cross-linking to enzyme and thus inactivating it, as has been described for mitomycin C at physiological pH (Siegel et al, 1992). In control incubations in which either drug, cofactor or enzyme were omitted, only background levels of damage were observed, showing that a complete activating system is required to convert EO9 to a bifunctional alkylating species. After reductive activation, EO9 was found to cross-link plasmid DNA more efficiently than an equivalent concentration of the bifunctional alkylating agent chlorambucil. Thus, these experiments have confirmed that purified DT-D catalysed reduction of EO9 results in activation of the compound to a difunctional alkylating species capable of cross-linking plasmid DNA. In contrast, Phillips and co-workers (Phillips et al, 1996) did not observe DNA cross-links after activation of EO9 by human DT-D using the same assay. The reason for these differing results is unknown but is likely to be due to kinetic differences between the human and the rodent enzyme.

The specificity of drug binding to particular sequences in DNA may be relevant to anti-tumour activity. Understanding this selectivity may permit rational design of compounds to target specific sequences of nucleotides. The *Taq* polymerase stop assay was used to examine the sequence selectivity of covalent modification to DNA by EO9.

In contrast to the cross-linking results, DNA alkylation by EO9 was observed in the absence of activation. It is likely that these covalent adducts are due to monofunctional alkylation through the aziridine moiety. Aziridines are highly reactive groups that can readily bind to nucleophiles. Binding appears to occur predominantly at guanine residues, which is consistent with results obtained for other aziridine-containing compounds (Mattes et al, 1986; Lee et al, 1992). This may be explained by the suggestion that alkylation is related to the electrostatic potential of guanine N-7 (Kohn et al, 1987). Electrostatic potential differs according to the base adjacent to the guanine, with the most negative bases being located in runs of guanines (Pullman and Pullman, 1981). Interestingly, the DNA alkylation binding pattern seen with EO9 is distinct from that of chlorambucil. In particular, the overall amount of binding of EO9 to guanine residues was reduced while some bases, such as position 527 (Figure 6, Lane E), were alkylated more strongly by EO9 than chlorambucil. It has been reported (Kohn et al, 1987) that the non-alkylating portion of the molecule can influence the sequence selectivity of alkylation.

When DT-D was present in the reaction, a decreased banding intensity was observed, which may be as a result of drug binding to the enzyme protein. Nevertheless, in the presence of a complete enzyme-activating system, the pattern of DNA modification induced by EO9 was distinctly different to that obtained by direct monofunctional alkylation. After enzyme activation, the compound appeared even more selective, binding to fewer and different sequences than when unactivated. A change in sequence selectivity after activation by DT-D has been reported previously for the aziridinyl benzoquinones Methyl DZQ and DZQ (Lee et al, 1992).

It is possible that one or both of the two hydroxyl leaving groups may be activated to alkylating moieties after enzymatic reduction. These together could lead to cross-link induction. Alternatively, either one of these could potentially be involved in cross-link formation, with the activated aziridine group forming the second arm of the cross-link. Activation to a bifunctional alkylating agent may result in drug being able to cause cross-links at certain sequences only and thus restricting lesions to fewer and more specific sites. A further complication may exist in the fact that these concentrations of reaction constituents would be expected to generate damaging oxygen radical species that have been shown to induce strand breaks in DNA under aerobic conditions (Bailey et al, 1993; Walton et al, 1991, 1992*a*). Strand breaks could potentially shorten the fragments of DNA and thus give artifactual results mimicking those of a covalent lesion.

In summary, there is good evidence that DNA damage is involved in the mechanism of cytotoxicity of EO9. We have shown for the first time that DNA damage is induced in intact cells after treatment with EO9 at relevant cytotoxic concentrations of the drug with both DNA strand breaks and interstrand cross-links being observed. Purified DT-D can activate EO9 to a species capable of inducing DNA interstrand cross-links. In contrast to DNA cross-linking, monofunctional alkylation by EO9 occurs in the absence of enzyme activation. However, the pattern of DNA sequence selectivity is different to that seen after reduction catalysed by DT-D. It is also clear that DNA interstrand cross-links can be formed under hypoxic conditions in cell lines lacking functional DT-D, thereby explaining the interesting cell cytotoxicity results (Plumb and Workman, 1994) and indicating the involvement of other enzymes in activation of EO9 to a DNA-damaging species under hypoxic conditions. The role played by various enzymes in activation of EO9 in the intact cell is likely to vary depending on their levels and affinities for EO9 as well as the levels of hypoxia (Workman, 1994). The precise identity of the cytotoxic, DNA-damaging species remains undetermined.

The present results may be of use in the interpretation of the clinical trial data with EO9 and in the design of future clinical studies, as well as in the development of new bioreductive agents of this type.

### ACKNOWLEDGEMENTS

We would like to thank the Cancer Research Campaign for supporting this work. Paul Workman also acknowledges the award of a Cancer Research Campaign Life Fellowship.

### REFERENCES

Bailey SM, Suggett N, Walton MI and Workman P (1992) Structure-activity relationships for DT-diaphorase reduction of hypoxic cell directed agents: indoloquinones and diaziridinyl benzoquinones. *Int J Radiat Oncol Biol Phys* **22:** 649–653

Bailey SM, Lewis AD, Patterson LH, Fisher GR and Workman P (1993) Free radical generation following reduction of EO9: involvement in cytotoxicity. *Br J Cancer* **67:** 8

Bailey SM, Wyatt MD, Lewis AD, Hartley JA and Workman P (1994*a*) Involvement of DT-diaphorase in the DNA cross-linking and sequence selectivity of the novel indoloquinone antitumour agent EO9. *Proc Am Assoc Cancer Res* **35:** 384

Bailey SM, Lewis AD and Workman P (1994*b*) Involvement of NADPH: cytochrome P450 reductase in activation of EO9 to a DNA damaging species. *Br J Cancer* **69:** 57

Chen S, Knox R, Lewis AD, Friedlos F, Workman P, Deng PSK, Fung M, Ebenstein D, Wu K and Tsai T (1995) Catalytic properties of NAD(P)H:quinone acceptor oxidoreductase. Study involving mouse, rat, human, and mouse-rat chimeric enzymes. *Mol Pharmacol* **47:** 934–939

Collard J, Matthew AM, Double JA and Bibby MC (1995) EO9: relationship between DT-diaphorase and response *in vitro* and *in vivo*. *Br J Cancer* **71:** 1199–1203

Fitzsimmons SA, Workman P, Grever M, Paull K, Camalier R and Lewis AD (1996) Reductase enzyme expression across the National-Cancer-Institute tumor cell line panel – correlation with sensitivity to mitomycin C and EO9. *J Natl Cancer Inst* **88:** 259–269

Gustafson DL, Beall HD, Bolton EM, Ross D and Waldren CA (1996) Expression of human NAD(P)H:quinone oxidoreductase (DT-diaphorase) in Chinese hamster

 © Cancer Research Campaign 1997

ovary cells: effect on the toxicity of antitumor quinones. *Mol Pharmacol* **50:** 728–735

Hartley JA, Berardini MD and Souhami RL (1991) An agarose gel method for the determination of DNA interstrand crosslinking applicable to the measurement of the rate of total and "second-arm" crosslink reactions. *Anal Biochem* **193:** 131–134

Hendriks HR, Pizao PE, Berger DP, Kooistra KL, Bibby MC, Boven E, Dreef-van der Meulen HC, Henrar REC, Fiebig HH, Double JA, Hornstra HW, Pinedo HM, Workman P and Schwartsmann G (1993) EO9: a novel bioreductive alkylating indoloquinone with preferential solid tumour activity and lack of bone marrow toxicity in preclinical models. *Eur J Cancer* **29A:** 897–906

Knox RJ, Friedlos F, Jarman M and Roberts JJ (1988) A new cytotoxic, DNA interstrand crosslinking agent, 5-(aziridni-1-yl)-4-hydroxylamino-2-nitrobenzamide, is formed from 5-(aziridin-1-ly)-2,4-dinitrobenzamide (CB 1954) by a nitroreductase enzyme in Walker carcinoma cells. *Biochem Pharmacol* **37:** 4661–4669

Knox RJ, Lydall DA, Friedlos F, Basham C, Rawlings CJ and Roberts JJ (1991) The Walker 256 carcinoma: a cell type inherently sensitive only to those difunctional agents that can form DNA interstrand crosslinks. *Mutat Res* **255:** 227–240

Kohn KW, Hartley JA and Mattes WB (1987) Mechanisms of DNA sequence selective alkylation of guanine N-7 positions by nitrogen mustards. *Nucleic Acids Res* **14:** 10531–10549

Lee C-S, Hartley JA, Berardini MD, Butler J, Siegel D, Ross D and Gibson NW (1992) Alteration in DNA cross-linking and sequence selectivity of a series of aziridinylbenzoquinones after enzymatic reduction by DT-diaphorase. *Biochemistry* **31:** 3019–3025

Maliepaard M, Wolfs A, Groot SE, de Mol NJ and Janssen LHM (1995) Indoloquinone EO9: DNA interstrand cross-linking upon reduction by DT-diaphorase or xanthine oxidase. *Br J Cancer* **71:** 836–839

Mattes WB, Hartley JA and Kohn KW (1986) DNA sequence selectivity of guanine N7 alkylation by nitrogen mustards. *Nucleic Acid Res* **14:** 2971–2987

Oostveen EA and Speckamp WN (1987) Mitomycin analogues I. Indoloquinones as (potential) bisalkylating agents. *Tetrahedron* **43:** 255–262

Paull K, Camalier R, Fitzsimmons SA, Lewis AD, Workman P and Grever M (1994) Correlations of DT-diaphorase expression with cell sensitivity data obtained from the NCI human tumour cell line panel. *Proc Am Assoc Cancer Res* **35:** 369

Pavlidis N, Hanauske AR, Gamucci T, Smyth J, Lehnert M, Tevelde A and Lan J (1996) A randomized phase-II study with 2 schedules of the novel indoloquinone-EO9 in non-small-cell lung-cancer – a study of the EORTC early clinical-studies group (ecsg). *Ann Oncol* **7:** 529–531

Phillips RM (1996) Bioreductive activation of a series of analogs of 5-aziridinly-3 hydroxymethyl-1-methyl-2-[1h-indole-4,7-dione] prop-β-en-α-ol (E09) by human DT-diaphorase. *Biochem Pharmacol* **52:** 1711–1718

Plumb JA and Workman P (1994) Unusually marked hypoxic sensitisation to indoloquinone EO9 and mitomycin C in a human colon-tumour cell line that lacks DT-diaphorase activity. *Int J Cancer* **56:** 134–139

Plumb JA, Gerritsen M, Milroy R, Thomson P and Workman P (1994*a*) Relative importance of DT-diaphorase and hypoxia in the bioactivation of E09 by human lung tumor cell lines. *Int J Radiat Oncol Biol Phys* **29:** 295–299

Plumb JA, Gerritsen M and Workman P (1994*b*) DT-diaphorase protects cells from the hypoxic cytotoxicity of indoloquinone EO9. *Br J Cancer* **70:** 1136–1143

Ponti M, Souhami RL, Fox BW and Hartley, JA (1991) DNA interstrand crosslinking and sequence selectivity of dimethanesulphonates *Br J Cancer* **63:** 743–747

Pullman A and Pullman B (1981) Molecular electrostatic potential of the nucleic acids. *Quart Rev Biophys* **14:** 289–380

Roberts JJ and Friedlos F (1987) Quantitiative estimation of cisplatin-induced DNA interstrand cross-links and their repair in mammalian cells: relationship to toxicity. *Pharmaceut Ther* **34:** 215–246

Roberts JJ, Marchbank T, Kotsaki-Kovatsi VP, Boland MP, Friedlos F and Knox RJ (1989) Caffeine, aminoimidazolecarboxamide and dicoumarol, inhibitors of NAD(P)H dehydrogenase (quinone) (DT-diaphorase), prevent both the cytotoxicity and DNA interstrand crosslinking produced by 5-(aziridin-1-yl)-2,4-dinitrobenzamide (CB 1954) in Walker Cells. *Biochem Pharmacol* **38:** 4137–4143

Robertson N, Haigh A, Adams G and Stratford LJ (1994) Factors affecting sensitivity to EO9 in rodent and human tumour cells *in vitro*: DT-diaphorase activity and hypoxia. *Eur J Cancer* **30A:** 1013–1019

Rosenoer VM, Mitchley BCV, Roe FJC and Connors TA (1966) Walker carcinoma 256 in study of anticancer agents. I. Method for simultaneous assessment of therapeutic value and toxicity. *Cancer Res (Cancer Chemotherapy Screening Reports)* **26:** 937–939

Schellens JHM, Planting AST, van Acker BAC, Loos WJ, de Boer-Dennert M, van der Burg MEL, Koier I, Krediet RT, Stoter G and Verweij J (1994) Phase I and pharmacologic study of the novel indoloquinone bioreductive alkylating cytotoxic drug EO9. *J Natl Cancer Inst* **86:** 906–912

Siegel D, Gibson NW, Preusch PC and Ross D (1990*a*) Metabolism of diaziquone by NAD(P)H: (quinone acceptor) oxidoreductase (DT-diaphorase): role in diaziquone-induced DNA damage and cytotoxicity in human colon carcinoma cells. *Cancer Res* **50:** 7293–7300

Siegel D, Gibson NW, Preusch PC and Ross D (1990*b*) Metabolism of mitomycin C by DT-diaphorase: role in mitomycin C-induced DNA damage and cytotoxicity in human colon carcinoma cells. *Cancer Res* **50:** 7483–489

Siegel D, Beall H, Senekowitsch C, Kasai M, Arai H, Gibson NW and Ross D (1992) Bioreductive activation of mitomycin C by DT-diaphorase. *Biochemistry* **31:** 7879–7885

Smitskamp-Wilms E, Peters GJ, Pinedo HM, van Ark-Otte J and Giaccone G (1994) Chemosensitivity to the indoloquinone EO9 is correlated with DT-diaphorase activity and its gene expression. *Biochem Pharmacol* **47:** 1325–1332

Walton MI, Smith PJ and Workman P (1991) The role of NAD(P)H: quinone reductase (EC 16992, DT-diaphorase) in the reductive activation of the novel indoloquinone antitumour agent EO9. *Cancer Commun* **3:** 199–206

Walton MI, Suggett N and Workman P (1992*a*) The role of human and rodent DT-diaphorase in the reductive metabolism of hypoxic cell cytotoxins. *Int J Radiat Oncol Biol Phys*: **22:** 643–648

Walton MI, Bibby MC, Double JA, Plumb JA and Workman P (1992*a*) DT-diaphorase activity correlates with sensitivity to the indoloquinone EO9 in mouse and human colon carcinomas. *Eur J Cancer* **28A:** 1597–1600

Wanders J, Pavlidis N, Gamucci T, Huinink WWT, Kirix L, Wolff I and Verweij J (1995) Phase-II studies with EO9 in breast, colorectal, gastric, pancreatic and NSCLC. *Eur J Cancer* **31:** 565–565

Workman P (1994) Enzyme-directed bioreductive drug development revisited: a commentary on recent progress and future prospects with emphasis on quinone anticancer agents and quinone metabolizing enzymes, particularly DT-diaphorase. *Oncol Res* **6:** 461–475

© Cancer Research Campaign 1997